# Situational diagnosis of plans for the surveillance, prevention and control of COVID-19 at work in the hydrocarbon sector

**DOI:** 10.47626/1679-4435-2022-843

**Published:** 2022-03-30

**Authors:** Miguel Angel Burgos-Flores, Kevin Jesús Mayma-Aguirre, Katherine Yauri-Condor, Luis Alberto Ormeño-Delgado, Jaime Rosales-Rimache

**Affiliations:** 1 Instituto Nacional de Salud, Centro Nacional de Salud Ocupacional y Protección del Ambiente para la Salud, Lima (Lima), Perú.

**Keywords:** hydrocarbons, occupational health, public policy, COVID-19

## Abstract

**Introduction:**

During the COVID-19 pandemic in Peru, economic activities were restricted to limit the risk of contagion, and companies were required to prepare and register the plan for COVID-19 surveillance, prevention and control in the workplace prior to resuming activities.

**Objective:**

To describe the status of plan registration in the hydrocarbon sector during the first half of 2020, as well as the characteristics of health professionals and occupational health and safety supervisors or committees.

**Methods:**

Cross-sectional study with secondary analysis of databases obtained from the Integrated System for COVID-19 of Companies (Sistema Integrado para COVID-19 de Empresas, SISCOVID Empresas) and government public data on the mining sector.

**Results:**

We reviewed 2,566 plans and identified 54 198 hydrocarbon companies in the 2020 period. Regarding the plans registered, 5.9% of companies did not have an occupational safety and health supervisor or committee, and 63% do not have a health professional.

**Conclusions:**

There is evidence of non-compliance with plan registration requirements among hydrocarbon companies. There is also non-compliance with the requirement of having a health professional, occupational safety and health committee or supervisor. The findings show serious deficiencies in plan registration, which could lead to inadequate management of the activities to monitor, prevent and control COVID-19 in the workplace. Companies are advised to develop, register, and implement their plans to protect the health of their employees.

## Introduction

The novel coronavirus named *severe acute respiratory syndrome coronavirus 2* (SARS-CoV-2), which causes COVID-19, emerged in Wuhan, China, in November 2019, and pandemic was declared on March 11th, 2020. On June 29, 2020, the World Health Organization (WHO) reported 10,021,401 cases worldwide.^1,2^ In Peru, a state of health emergency was declared on March 16 of the same year, restricting economic activities only to essential sectors, including the hydrocarbon sector.^3^ Furthermore, a total of 285,213 cases of COVID-19 were reported in Peru at the end of June by the National Center of Epidemiology, Prevention and Control of Diseases of the Ministry of Health.^2,4^ Early responses to the pandemic have been logically essential to public health and to national economies, although knowledge was limited in the first months, which made evidence-based decision making more complex.^5,6^

The Peruvian state enacted health measures to reduce the risk of SARS-CoV-2 infection in the population, notably mandatory social isolation, social distancing measures, and use of masks^3^; at the labor level, the resumption of economic activities authorized by the State during the health emergency was developed in four stages. With the publication of DS no. 080-2020-PCM of May 3^rd^, 2020, there was the beginning of Stage I, which encompassed economic activities such as mining and industry, construction, services, and supply markets. On June 4^th^ of the same year, DS no. 101-2020-PCM opens Stage II, adding activities such as agriculture, small and medium mining companies, manufacturing, automotive trade, and a greater range of services; the hydrocarbon sector was totally covered in these two stages. The resumption of activities by companies was conditioned to the implementation of the technical document “Guidelines for surveillance, prevention, and control of health of workers at risk of exposure to COVID-19,” developed by the National Center of Occupational Health and Environmental Protection for Health (Centro Nacional de Salud Ocupacional y Protección del Ambiente para la Salud, CENSOPAS-INS), which establishes the creation, implementation, and registration of plans for COVID-19 surveillance, prevention and control at work (plans), by companies of the authorized sectors.^7-9^ The CENSOPAS-INS was in charge of managing the plan registration system that grants the right to reopen authorized economic activities.^10^

The technical document addressed the following guidelines: cleaning and disinfection of work centers, assessment of workers’ health condition, hand washing and disinfection, awareness of contagion prevention, preventive measures of collective implementation to maintain environments appropriately ventilated with cyclic renovation of the air, social distancing among workers, etc., use of personal protective equipment according to the level of risk of exposure to COVID-19, surveillance of other comorbidities related to the pandemic context; furthermore, the document indicates provisions for the completion of plan registration in the Integrated System for COVID-19 of Companies (Sistema Integrado para COVID-19 de Empresas, SISCOVID Empresas) and for the execution and supervision of the plan by health care professionals and an Occupational Safety and Health (OSH) supervisor or committee, respectively.^9^

The hydrocarbon sector is considered one of the most affected economic sectors, with a decrease from 12% to 19% in its production, leading to economic consequences associated with the COVID-19 pandemic in Peru; moreover, this sector accounts for nearly 3% of the country’s gross domestic product (GDP).^11^ Since the hydrocarbon sector was considered in the first stages of economic resumption during the health emergency caused by COVID-19, workers were understandably at higher risk of exposure to COVID-19 compared to those working in other extractive and service sectors.^4,7^

The present study aimed to describe the status of plan registrations during the first and second stages of economic resumption in the hydrocarbon sector and the characteristics of occupational health services and OSH supervisors or committees. We believe that our results are relevant to improve the level of implementation of OHS policies in the context of COVID-19, with the participation of health care professionals and OSH representatives established by provisions for the execution and supervision of the plans.

## Methods

### Study design

Cross-sectional study with secondary analysis of two databases: (1) list of companies operating in the hydrocarbon sector in 2020, obtained from the online portal of the Supervisory Agency for Investment in Energy and Mining (Organismo Supervisor de la Inversión en Energía y Minería, OSINERGMIN),^12^ and (2) plan registrations from hydrocarbon companies on the SISCOVID Empresas^13^ platform of the Ministry of Health that took place from May 9^th^ to June 30^th^, 2020, the period corresponding to the first and second stages of economic resumption.

### Population

A total of 54,198 hydrocarbon companies were identified on the OSINERGMIN database (including offices and projects), of which the subactivity “Carriers of liquid fuel and OHDP by truck” accounted for the greatest percentage (23.9%), followed by the subactivities “Direct consumers of LPG” and “Points of sale of LPG,” with 18.7% and 18.4% respectively (Table 1). With regard to the region of company’s offices, the greatest percentage are located in Lima (34.4%), followed by Arequipa (8.5%).

**Table 1:** Description of activities of the registered companies operating in the hydrocarbon sector

Subsector	n	%
Carriers of LF and OHDP by truck	12,953	23.9
Direct consumers of LPG	10,125	18.7
Points of sale of LPG	9,952	18.4
Distributors of LPG cylinders	4,979	9.2
Gas stations and services stations	3,142	5.8
Carriers of LPG in cylinders	3,084	5.7
DCLF and OHDP with fixed installations	1,834	3.4
Retail distributors of LF and OHDP	1,303	2.4
Others	6,826	12.5

CDCL = direct consumers of liquid fuel; LF = liquid fuel; LPG = liquefied petroleum gas; OHDP = other hydrocarbon-derived products.

Source: Supervisory Agency for Investment in Energy and Mining (Organismo Supervisor de la Inversión en Energía y Minería).

The search on the database of SISCOVID Empresas identified 2,566 plans with approved registration during the first and second stages of economic resumption, corresponding to formal companies of the different activities of the hydrocarbon sector.

The sample was census-based. Exclusion criteria consisted of plan registrations with rejected status or that were not concluded.

### Techniques and procedures

In order to supplement data from hydrocarbon companies in Peru, information was requested to OSINERGMIN, which provided the link https://www.osinergmin.gob.pe/empresas/hidrocarburos/Paginas/RegistroHidrocarburos/registros-habiles-informacion-historica.htm, where data on Taxpayer Identification Number, Region, and Subactivity were retrieved for all registered companies operating in the hydrocarbon sector on September 30^th^, 2020.^12^

Plan registrations from the hydrocarbon sector were fully retrieved on March 15^th^, 2021, from the restricted access database at https://saludtrabajo.minsa.gob.pe/page/homepage, as known as SISCOVID Empresas,^13^ which was accessed with approval of CENSOPAS-INS. This online platform allowed companies to create a username and a password to finally register their plan. This platform provided the following information: Region of origin, Number of employees, Health care professional, OSH Committee or Supervisor, Registration Status, Budget.

The plan registration process involved two stages: (1) submission of the plan to Ministry of Energy and Mining, for authorization of resumption of economic activity and subsequent registration on SISCOVID Empresas, and (2) automatic registration through a 24-step form directly on SISCOVID Empresas.

Database was generated on Microsoft Excel®, considering quality criteria such as: duplicate entry, random review of records, and data cleaning processes (including identification of divergent data, atypical values, revision in the source).

### Data analysis

Data were presented through descriptive statistics as absolute and relative frequencies, and using contingency tables. Furthermore, median and interquartile range were employed, as well as minimum and maximum values. Statistical analysis was conducted using the STATA statistical package, version 15.0.

### Ethical considerations

Since this study is based on secondary databases, it was not considered for submission for approval by an ethics committee.

## Results

Overall, 2,570 plan registrations of the hydrocarbon sector were retrieved from the SISCOVID Empresas database, of which four were excluded based on exclusion criteria; therefore, 2,566 registrations were considered for statistical analysis. Of the latter, 895 plans (34.9%) were registered in the first step of registration, and 1,671 (65.1%) in the automated stage. The budget allocated to the implementation of the plan was not reported in 19.7% of the plans. As for the region where the company’s headquarters are located, Lima accounts for 43% of the cases, followed by Arequipa, with 7%. Table 1 shows the distribution of the number of employees per company, revealing that companies with 1 to 20 employees have the highest proportion of cases, accounting for 89.1% of them (Table 2).

**Table 2:** Distribution of plans registered on the Integrated System for COVID-19 of Companies (*Sistema Integrado para COVID-19 de Empresas*) in the hydrocarbon sector

Characteristic of the plan	n	%
Region		
Lima	1,104	43.0
Others	1,462	57.0
Health care professionals		
No	1,616	63.0
Yes	950	37.0
Type of professional		
Physician	875	34.1
Nurse	39	1.5
Both	36	1.4
Responsible for supervising OSH		
Not reported	151	5.9
Supervisor	1,979	77.1
Committee	436	17.0
No. of employees	Med: 6; Min: 0; Max: 3218; IQR: 9	
1 to 20	2,286	89.1
21 to 50	100	3.9
51 to 100	80	3.1
101 to 500	77	3.0
More than 500	19	0.7
Not reported	4	0.2
Declared budget		
No	502	19.6
Yes	2,064	80.4

IQR = interquartile range; Max = max; Med = median; Min = minimum; OHS = occupational health and safety.

Source: Integrated System for COVID-19 of Companies (Sistema Integrado para COVID-19 de Empresas).

With regard to health care professionals, records indicate that 63% (1,616) of registered companies did not have a health care professional. In the case of companies with 101 to 500 and with more than 500 employees, 36.9% and 26.7% had a medical professional and a nursing professional, respectively (Table 3). As for compliance with the OHS regulation related to plan supervision, it was observed that 5.9% (151) of registrations did not report to have an OHS supervisor or committee (Table 3).

**Table 3:** Distribution of plan supervision and health care professionals according to the number of employees

Characteristic of OHS	Number of employees, n (%)					
	1 to 20	21 to 50	51 to 100	101 to 500	More than 500	Not reported
Health care professionals						
No	1 508 (66.0%)	46 (46.0%)	42 (52.5%)	12 (15.6%)	4 (21.1%)	4 (100.0%)
Si	778 (34.0%)	54 (54.0%)	38 (47.5%)	65 (84.4%)	15 (78.9%)	0 (0.0%)
**Type of professional**						
Nurse	24 (3.1%)	13 (24.1%)	2 (5.3%)	0 (0.0%)	0 (0.0%)	0 (0.0%)
Physician	750 (96.4%)	39 (72.2%)	34 (89.4%)	41 (63.1%)	11 (73.3%)	0 (0.0%)
Both	4 (0.5%)	2 (3.7%)	2 (5.3%)	24 (36.9%)	4 (26.7%)	0 (0.0%)
**Responsible for supervising OHS**						
Not reported	139 (6.1%)	8 (8.0%)	2 (2.5%)	1 (1.3%)	0 (0.0%)	1 (25.0%)
Supervisor	1924 (84.2%)	18 (18.0%)	16 (20.0 %)	14 (18.2%)	5 (26.3%)	2 (50.0%)
Committee	223 (9.7%)	74 (74.0%)	62 (77.5%)	62 (80.5%)	14 (73.7%)	1 (25.0%)

OHS = Occupational Health and Safety.

Source: Integrated System for COVID-19 of Companies (Sistema Integrado para COVID-19 de Empresas).

An analysis of plan registrations according to the region of the registered companies that were granted permission to operate by the OSINERGMIN showed that the highest and the lowest rate of compliance were found in the regions of Huancavelica (14.4%) and Puno (1.7%), respectively (Table 4).

**Table 4:** Distribution of plan registrations and hydrocarbon companies according to region

Region	Hydrocarbon companies		Plan registration		% of registrations*
	n	%	n	%	
Amazonas	436	0.8	55	2.1	12.6
Ancash	1,291	2.4	59	2.3	4.6
Apurímac	550	1.0	21	0.8	3.8
Arequipa	4,609	8.5	179	7.0	3.9
Ayacucho	607	1.1	73	2.8	12.0
Cajamarca	1,623	3.0	80	3.1	4.9
Cusco	2,949	5.4	113	4.4	3.8
Huancavelica	215	0.4	31	1.2	14.4
Huánuco	850	1.6	35	1.4	4.1
Ica	1,636	3.0	41	1.6	2.5
Junín	1,711	3.2	112	4.4	6.5
La Libertad	3,753	6.9	100	3.9	2.7
Lambayeque	1,957	3.6	83	3.2	4.2
Lima	18,665	34.4	1,104	43.0	5.9
Loreto	898	1.7	63	2.5	7.0
Madre de Dios	732	1.4	62	2.4	8.5
Moquegua	970	1.8	23	0.9	2.4
Pasco	330	0.6	12	0.5	3.6
Piura	1,782	3.3	73	2.8	4.1
P.C. Callao	1,724	3.2	43	1.7	2.5
Puno	3,575	6.6	59	2.3	1.7
San Martin	1,785	3.3	55	2.1	3.1
Tacna	558	1.0	24	0.9	4.3
Tumbes	100	0.2	7	0.3	7.0
Ucayali	892	1.6	59	2.3	6.6

* Percentage of registrations: (total of plan registrations in the region × 100)/Number of companies in the region.

Source: Supervisory Agency for Investment in Energy and Mining (Organismo Supervisor de la Inversión en Energía y Minería) and Integrated System for COVID-19 of Companies (Sistema Integrado para COVID-19 de Empresas).

## Discussion

A total of 2,566 plans were registered by hydrocarbon companies from May to June 2020. When the number of records was compared with the total hydrocarbon companies (offices and projects) authorized in 2020, a low percentage of plan registrations from the sector was observed in stages 1 and 2 of economic resumption.^12^ Therefore, it was found that most companies of this sector did not implement biosafety measures to prevent COVID-19 contagion at work and that their personnel were unaware of their risks of exposure. Moreover, there was a lack of procedures for symptom surveillance before and after identification of positive cases in the workplace, which represents a challenge for COVID-19 prevention.^14^

It was evidenced that the implementation of the plan in a company of the mining sector reached 96% of compliance and allowed for resumption of labor activities with a minimum of cases of COVID-19, which had decreased throughout the five month following the implementation of the seven guidelines described in the plan.^15^ Another study conducted in the fishing sector showed that the implementation of a plan promoted a safe work environment, by measuring the variation in the decrease of cases of COVID-19 in the areas of production and storage, which accounted for the highest number of employees.^16^

The analysis of hydrocarbon companies according to region showed that Lima and Callao had the highest number of companies (20,389), although the percentage of compliance with requirement of having an approved plan registration was 5.6% in these regions.^12^ Furthermore, on June 29, 2020 the Ministry of Health reported that 45.7% of positive cases of COVID-19 were found in the region of Lima and Callao, which suggests a possible relationship with compliance with plan registration.^4^

Law no. 29783 establishes that companies with up to 20 employees should have an OHS supervisor; similarly, those with more than 20 employees should have an OHS committee, which is responsible for approving the plan and performing an internal surveillance of its compliance.^9,17^ However, of the companies with approved plans, 5.9% did not have an OHS supervisor or committee. Furthermore, workers in the hydrocarbon sector are entitled to a complementary occupational hazard insurance and, consequently, all companies operating in this sector should have at least one health care professional; however, it was observed that 63% of companies with a registered plan did not have a health care professional.^9^

As for South American countries, the measures taken by the government of Argentina,^18^ Brazil^19^ and Chile^20^ were similar to those implemented in Peru, enacting administrative measures that had limited non-essential economic activities and regulated essential economic activities, with the purpose of preventing and reducing COVID-19 contagion in the workplace.

In Argentina, the action protocols for COVID-19 prevention and control according to economic sector established the implementation of five general guidelines of COVID-19 prevention, including measures to be implemented in the work environment.^21^ In Brazil, COVID-19 prevention measures in the workplace were established through ordinance, including general measures, such as actions to be taken in cases of suspected and confirmed COVID-19 and their contacts, respiratory etiquette, social distancing, cleaning and disinfection of hands and environments, use of personal protective equipment, and characterization of workers at risk.^22^ In Chile, the national protocol “COVID Way of Living” (“Modo COVID de Vida”) was established, being a part of the “Step by Step Labor Plan” (“Paso a Paso Laboral”) program, which proposes seven guidelines that consist of informing, organizing and generating agreements, socializing and qualifying, adapting and implementing, prioritizing mental health, among others.^23^ These guidelines may vary among each other in certain aspects; however, they have the same goal: preventing and controlling COVID-19 in the workplace.^24^

One of the limitations of this study was not conducting a registration of hydrocarbon companies that performed economic activities during the stages 1 and 2 of resumption. Likewise, the present study assessed the registration process of the plans but did not assess the level of implementation of these plans through a compliance audit or other mechanism of in situ assessment; moreover, the incidence of cases of COVID-19 in each company or in the sector for the study period was not assessed, since this information was not reported by the OSINERGMIN or other public entity relevant to the hydrocarbon sector.

## Conclusions

Finally, we concluded that the State policies adopted by the Peruvian government have been necessary in a pandemic situation, in which workers’ health is prioritized for the reopening of economic activities. Hydrocarbon companies were included in the first two stages of economic resumption. However, it was observed that most of these companies did not comply with the requirement of plan registration, as well as in the declaration of having a health care professional in their staff and an OHS committee or supervisor. Our findings reveal serious deficiencies in plan registration, which could lead to inadequate management of the activities to monitor, prevent and control COVID-19 in the workplace. Companies are advised to develop, register, and implement their plan to protect the health of their employees, since this plan is an important document of OHS management; moreover, it allows for OHS committees and supervisors or employees to monitor the implementation of the guidelines described in the plan.

## References

[1] Zhu N, Zhang D, Wang W, Li X, Yang B, Song J (2020). A novel coronavirus from patients with pneumonia in China,
2019. N Engl J Med.

[2] World Health Organization (2020). Coronavirus disease (COVID-19) - Situation Report - 161
[Internet].

[3] El Peruano (2020). Decreto Supremo que declara Estado de Emergencia Nacional por las graves
circunstancias que afectan la vida de la Nación a consecuencia del brote del
COVID-19 [Internet].

[4] Perú, Ministerio de Salud (2020). SITUACION ACTUAL “COVID-19“ al 29 de junio 2020.

[5] Ionnidis JPA. (2020). A fiasco in the making? As the coronavirus pandemic takes hold, we are
making decisions without reliable data [Internet].

[6] Lipsitch M. (2020). Social distancing is a good place to start [Internet].

[7] El Peruano (2020). Decreto Supremo que aprueba la reanudación de actividades económicas en
forma gradual y progresiva dentro del marco de la declaratoria de Emergencia
Sanitaria Nacional por las graves circunstancias que afectan la vida de la
Nación a consecuencia del COVID-19 [Internet].

[8] El Peruano (2020). Decreto Supremo que aprueba la Fase 2 de la Reanudación de Actividades
Económicas dentro del marco de la declaratoria de Emergencia Sanitaria
Nacional por las graves circunstancias que afectan la vida de la Nación a
consecuencia del COVID-19, y modifica el Decreto Supremo Nº 080-2020-PCM
[Internet].

[9] El Peruano (2020). Aprueban el Documento Técnico “Lineamientos para la vigilancia de la
salud de los trabajadores con riesgo de exposición a COVID-19”
[Internet].

[10] El Peruano (2020). Delegan en el Instituto Nacional de Salud, a través del Centro Nacional
de Salud Ocupacional y Protección del Ambiente para la Salud (CENSOPAS), la
administración del registro del “Plan para la vigilancia, prevención y
control de COVID-19 en el trabajo” en el Sistema Integrado para COVID-19
(SISCOVID-19) del Ministerio de Salud; así como su fiscalización posterior
[Internet].

[11] Gestión (2020). Los hidrocarburos en el Perú: su impacto en la economía nacional y
regional [Internet].

[12] Perú, Organismo Supervisor de la Inversión en Energia y
Mineria (2020). Información histórica de Registros Hábiles [Internet].

[13] Perú, Ministerio de Salud (2020). Homepage [Internet].

[14] Lai CC, Shih TP, Ko WC, Tang HJ, Hsueh PR. (2020). Severe acute respiratory syndrome coronavirus 2 (SARS-CoV-2) and
coronavirus disease-2019 (COVID-19): the epidemic and the
challenges. Int J Antimicrob Agents.

[15] Huaracayo Perez W. (2021). Implementación de un plan de vigilancia para el control y prevención
frente al SARS-CoV2 en la Unidad Minera Las Águilas CIEMSA-2020
[tesis].

[16] Yanet PQR, Laritza ROJ, Smith SNVL. (2020). Implementación de un plan de vigilancia, prevención y control de
Covid-19 en el trabajo en la empresa Océano Seafood S.A. - Paita.

[17] El Peruano (2011). Ley de seguridad y salud en el trabajo y su modificatoria
[Internet].

[18] COVID-19 Observatory in Latin America and Caribbean (2020). Follow-up of the evolution of COVID-19 measures - Argentina
[Internet].

[19] COVID-19 Observatory in Latin America and Caribbean (2020). Follow-up of the evolution of COVID-19 measures - Brazil
[Internet].

[20] COVID-19 Observatory in Latin America and Caribbean (2020). Follow-up of the evolution of COVID-19 measures - Chile
[Internet].

[21] Argentina, Ministerio de Salud (2020). Protocolos y recomendaciones [Internet].

[22] Brasil, Imprensa Nacional (2020). Portaria conjunta no 19, de 18 de junho de 2020 [Internet].

[23] Chile, Instituto de Seguridad Laboral (2020). Ministerio del Trabajo publica “Paso a Paso Laboral”: Hoja de ruta
enfocada en trabajadores y empleadores [Internet].

[24] Organización de los Estados Americanos (2020). Guías y protocolos de desconfinamiento y vuelta al trabajo
[Internet].

